# Relationships between animal management and habitat characteristics with two potential indicators of welfare for bottlenose dolphins under professional care

**DOI:** 10.1371/journal.pone.0252861

**Published:** 2021-08-30

**Authors:** Lance J. Miller, Lisa K. Lauderdale, Jill D. Mellen, Michael T. Walsh, Douglas A. Granger

**Affiliations:** 1 Conservation Science and Animal Welfare Research, Chicago Zoological Society–Brookfield Zoo, Brookfield, IL, United States of America; 2 Biology Department, Portland State University, Portland, OR, United States of America; 3 Department of Comparative, Diagnostic & Population Medicine, College of Veterinary Medicine, University of Florida, Gainesville, FL, United States of America; 4 Institute for interdisciplinary Salivary Bioscience Research, University of California, Irvine, CA, United States of America; Institute of Deep-sea Science and Engineering, Chinese Academy of Sciences, CHINA

## Abstract

Accredited zoos and aquariums continually strive to ensure high levels of animal welfare for the animals under their professional care. Best management practices include conducting research to better understand factors that lead to optimal welfare and then turning findings into practice. The current study is part of the larger Cetacean Welfare Study or more formally, “Towards understanding the welfare of cetaceans in zoos and aquariums.” Facilities participating in the study were accredited by the Alliance of Marine Mammal Parks and Aquariums and/or the Association of Zoos and Aquariums. Animal management factors and habitat characteristics were examined in relation to two potential indicators of welfare for common (*Tursiops truncatus*) and Indo-Pacific (*Tursiops aduncus*) bottlenose dolphins. Specifically, we examined environmental enrichment, animal training, and habitat characteristics that were significantly related to behavioral diversity and route tracing, a form of stereotypic behavior. Behavior was recorded from 47 animals at 25 facilities around the world. Overall, the rate of route tracing behavior observed during the study was very low and few animal management factors or habitat characteristics were found to be related to this behavior. One factor, enrichment diversity, had a significant positive relationship with route tracing and an inverse relationship with behavioral diversity. This finding may be a product of a response mounted by animal care specialists to the behavior as opposed to a cause. Animals that engaged in this behavior were likely provided more diverse enrichment in attempts to ameliorate the stereotypic behavior. However, multiple factors were found to significantly relate to behavioral diversity, a potential positive indicator of welfare for bottlenose dolphins. Dolphins that were trained on a predictable schedule had higher behavioral diversity than those on a semi-predictable schedule. There was a positive significant relationship between behavioral diversity and the number of habitats to which an animal had access, and a significant inverse relationship with the maximum depth of the habitat. Finally, animals that were split into groups and reunited or rotated between subgroups had higher behavioral diversity than animals managed in the same group. Information gained from the current study suggested that animal management techniques may be more important in ensuring good welfare for bottlenose dolphins than focusing on habitat size.

## Introduction

Evaluation of factors that lead to optimal welfare for animals in zoos and aquariums is critical. This includes examining both input-based factors (resource-based) as well as how those factors impact outputs (animal-based indicators of animal welfare) [1]. There are many factors (inputs) that can influence welfare of animals under professional care, but they can generally be categorized into three groups. These include the animal’s natural history, individual history, and management. Within a zoological facility, understanding how animal management factors influence welfare is probably most important as management can typically be changed to impact welfare. Examples of animal management inputs that have been found to impact the welfare of animals include environmental enrichment, animal training, and habitat characteristics [2, 3]. All three of these inputs are likely important in different ways to the welfare of bottlenose dolphins under professional care in zoos and aquariums.

Environmental enrichment can be defined as “an animal husbandry principle that seeks to enhance the quality of captive animal care by identifying and providing environmental stimuli necessary for optimal psychological and physiological well-being” [4]. As a complex, social species, it would be anticipated that an enrichment program would be critical for dolphins’ welfare. Previous research has demonstrated that having a diverse enrichment program, or providing a variety of different types of enrichment, was correlated with indicators of welfare for African elephants (*Loxodonta africana*), another complex and social species [5]. Research specifically with bottlenose dolphins found that the timing of the enrichment is just as important as the type of enrichment [6]. Another study examining object enrichment with common bottlenose dolphins (*Tursiops truncatus*) found that only 50% of the objects provided elicited the desired behavioral goal [7]. This demonstrates the importance of setting goals as well as documenting, evaluating, and readjusting an enrichment program to ensure the behavioral needs of animals are met [8, 9]. Finally, it has also been suggested that the positive reinforcement training used for educational programs (i.e., dolphin presentations and interaction programs) may be a form of enrichment for bottlenose dolphins [10]. Dolphins had higher levels of behavioral diversity following the educational programs compared to non-program times, thought to be a potential positive indicator of animal welfare [11–13]. In addition, play behavior was higher following programs compared to non-program times, with play thought to be a potential indicator of good welfare although there are some mixed reviews [14, 15].

Positive reinforcement training is a common management practice used with bottlenose dolphins [16]. Positive reinforcement training is the process of rewarding behavior with the goal of increasing the occurrence of that behavior in the future [17]. One of the goals of training is for the animals to learn to voluntarily participate in their own health care and acclimate to new experiences [16]. The training process can also be used to teach animals to participate in educational programs such as dolphin presentations or interaction programs [18]. As previously noted, research has also demonstrated that these programs are likely enriching for the animals [10]. Similarly, another study examining interaction programs found a short-term increase in play behavior following the programs [19]. Although the study design was different, a final study examining interaction programs found no detrimental effects of the programs, but dolphins did use a refuge area significantly more during the programs [20]. While all the benefits of positive reinforcement training for bottlenose dolphins are not known, research with other socially intelligent species (e.g., primates) has demonstrated the benefits of positive reinforcement training on the welfare of animals [21, 22].

Another management factor or input that likely impacts the welfare of bottlenose dolphins is habitat characteristics. Only a handful of studies have examined the impact of habitat characteristics on bottlenose dolphin behavior. For example, bottlenose dolphins in a larger habitat displayed higher activity levels than when in a smaller habitat [23]. Similarly, two studies showed that dolphins in open larger facilities (i.e., ocean pens) spent significantly more time swimming than dolphins in smaller closed facilities (i.e., non-ocean habitats) [24, 25]. Another study found similar results in that dolphins in larger open facilities spent more time swimming in a linear pattern than a circular pattern when compared to dolphins in smaller closed facilities [26]. In all three studies, dolphins spent less time floating in open larger facilities when compared to animals in smaller closed facilities [24–26]. In terms of depth, one study examining space use in dolphins found that dolphins utilized moderate depth locations in the habitat as opposed to shallow or deep areas [27]. While there are some confounding factors in terms of open facilities being larger and closed facilities being smaller, it appears that habitat characteristics can impact the behavior of dolphins. Additional research is clearly needed to better understand these impacts.

While bottlenose dolphins are a species typically thought to do well under professional care, zoos and aquariums are dedicated to continuous improvement [28]. Two potential indicators of animal welfare for bottlenose dolphins include repetitive behaviors and behavioral diversity. While still up for debate, repetitive or stereotypic behavior for the purpose of this study can be defined as any unvarying and functionless behavior [29]. Behavioral diversity can be defined as a measure of species-typical behavior incorporating both the frequency and richness [11]. Repetitive behavior is not always linked to situations of poor welfare but should always be examined to try and determine the cause [30]. While previous research has demonstrated that bottlenose dolphins engage in repetitive behavior at low levels under professional care, having a better understanding of factors that influence the behavior would be beneficial [10]. Importantly, the absence of negative indicators of animal welfare does not indicate that an individual animal is thriving [31]. While repetitive behavior can be a potential indicator of negative animal welfare, behavioral diversity may be a potential indicator of positive welfare. Previous research has demonstrated an inverse relationship between fecal glucocorticoid metabolites and behavioral diversity for several species including bottlenose dolphins [11–13]. Additionally, much research has shown an inverse relationship between repetitive behavior and behavioral diversity [32–35].

The goal of the current study was to determine how environmental enrichment, animal training, and habitat characteristics affect two potential indicators of animal welfare, repetitive behavior and behavioral diversity in common bottlenose dolphins and Indo-Pacific bottlenose dolphins. Information gained from this study can help facilities make informed animal management decisions to promote optimal welfare.

## Methods

### Ethics statement

Animal care and veterinary staff reviewed and approved the project at each participating facility. The study was also reviewed and approved by the U.S. Navy Marine Mammal Program Institutional Animal Care and Use Committee #123–2017.

### Subjects and facilities

Facilities accredited by the Alliance of Marine Mammal Parks and Aquariums (AMMPA) and/or Association of Zoos and Aquariums (AZA) that professionally managed one of the two focal subspecies were invited to participate. This study is part of a larger project entitled “Towards Understanding the Welfare of Cetaceans in Zoos and Aquariums”. The focal subspecies are common bottlenose dolphins and Indo-Pacific bottlenose dolphins. Sampling design was semi-random and counterbalanced to include two animals from each facility to have an approximately equal number of animals across both sexes and through a range of ages representing the variability across facilities. The result was a sample of 86 dolphins from 40 facilities. Six dolphins observed during the first five-week data collection period were not observed in the second five-week data collection period. Eight dolphins observed during the second five-week period were not observed during the first five-week period. There were also two dolphins observed that changed facilities between the two data collection periods. All dolphins observed are displayed in S1 Appendix including their age, sex, and total minutes visible.

### Data collection

Data were collected in two separate five-week periods. The first five-week data collection period was between July and November of 2018 and the second five-week data collection period occurred from January through April of 2019. Focal animals were video recorded three times a week for five weeks during each of the data collection periods. Focal animals were video recorded during one of three time periods including morning (8:00–11:00), mid-day (11:00–14:00) and afternoon (14:00–17:00). In order to ensure accurate identification of the focal animals, animal care staff or interns were requested to conduct the videotaping. The times selected were counterbalanced across days and times (Table 1). Staff and interns were instructed to videotape anytime within the selected time period. The only times they could not select to videotape were around a training session, research session, dolphin presentation or interaction program. Specifically, they were instructed not to film 20 minutes before or after any of those programs. The week prior to actual data collection, the animal care staff member or intern stood in the filming location during typical observation times to habituate the dolphins to an observer. The animal care staff member or intern was also prohibited from interacting (eye contact or engaging with) with the dolphins while filming. A piece of paper with an exhibit code, name of the dolphin, date, time, and identification and observation numbers was held up in front of the camera. Observations were 25 minutes in duration resulting in a total of 375 minutes of video during each month of observation. Polarized film was attached to a Fuji Film XP120 waterproof video camera to reduce glare. As all facilities do not have underwater viewing, all dolphins were recorded above the surface of the water to ensure data were comparable across facilities.

**Table 1:** Observation schedule used to videotape focal animals.

Week	Time Period	Monday	Tuesday	Wednesday	Thursday	Friday	Saturday	Sunday
1	8:00–11:00	D1			D2			
	11:00–14:00	D2		D1				
	14:00–17:00			D2	D1			
2	8:00–11:00	D2					D1	
	11:00–14:00						D2	D1
	14:00–17:00	D1						D2
3	8:00–11:00			D1			D2	
	11:00–14:00			D2	D1			
	14:00–17:00				D2		D1	
4	8:00–11:00			D2				D1
	11:00–14:00	D1						D2
	14:00–17:00	D2		D1				
5	8:00–11:00				D1			D2
	11:00–14:00				D2		D1	
	14:00–17:00						D2	D1

Note: D1 is Focal Dolphin 1 and D2 is Focal Dolphin 2.

Observers were trained and reliability between individuals of r > 0.80 was required before scoring actual videos. In total there were nine observers that scored all of the videos. A combination of instantaneous sampling (behavioral states) and continuous sampling (behavioral events) was used to score the video. While there were many behaviors collected for the larger study, the study described here focuses on the behavioral event of route tracing as a form of repetitive behavior and behavioral diversity. Route tracing was defined as a dolphin swimming in a fixed repetitive pattern using the same path to move from one point to another. The dolphin was required to complete the pattern three or more times to be scored as route tracing. Route tracing was converted to a rate by dividing by the total number of minutes visible. In order to calculate behavioral diversity, species-appropriate behaviors excluding stereotypic behavior and inactivity were selected for the calculation and are highlighted in Table 2 [12, 31]. Behavioral definitions from previous studies were adapted for the current study [10, 36–42]. Fluke-in dives and fluke-out dives were separated out due to their occurrence during different activities in the wild [43].

**Table 2:** Ethogram of behaviors used to calculate behavioral diversity.

Behavior	Definition
Fast Swim	Dolphin sustains a rapid speed, swimming in one direction, for more than 3 s, producing a wake at the surface (while not chasing another individual).
Fluke-In Dive	Dolphin surfaces and then dives down under the water with the fluke remaining below the surface of the water.
Fluke-Out Dive	Dolphin surfaces and then dives down under the water raising its fluke up in the air and out of the water.
Group Social Ball	Two or more dolphins swim around each other, often mouthing and chasing each other. This is often associated with sexual play. It is extremely difficult to identify the individual behaviors that each animal is doing.
Interact with Conspecific	Dolphin orients toward and mutually interacts with one or more conspecifics for more than 3 s.
Interact with Object	Dolphin interacts with an object which can include holding, carrying, balancing, or pushing the object; interactions will only be counted once if within 3 s of the previous interaction.
Jump/Breach	A large aerial locomotion in which all of the dolphin’s body comes completely out of the water.
Mount	One dolphin’s genital area touches another’s genital area.
Porpoise	Small bows usually performed several times in a row characterized by small forward motion leaps out of the water. The dolphin’s head re-enter the water as the tail is exiting the water.
Spy Hop	Dolphin moves in such a way that the upper part of the body rises above the water in a vertical position.
Tactile/Rub	Dolphin contacts or actively rubs another dolphin a manner that is not considered sexual contact.
Ventral Swim	Dolphin swims inverted with ventral side pointing towards the surface for more than 3 s.

The Shannon Diversity Index was utilized where behavioral rates observed are divided by total of all behavioral rates as p, the natural log as ln, and the sum across behavior as ∑ [44].


$$H=-\sum_{i=1}^{s}p_{i}\ln p_{i}$$

The Shannon Diversity Index is helpful when one factor (behavior) is dominant to detect subtle changes in all factors (behaviors) [45]. Behavioral diversity calculated utilizing the Shannon Diversity Index considers both the number of behaviors observed and the rate of occurrence [32].

While an attempt was made to reduce the reflection of the water with polarizing film on the camera, the glare at some facilities was too great and dolphins could not be viewed for extended periods of time. Additionally, some habitats were ocean pens and were subject to natural tides and currents which resulted in limited underwater visibility for extended periods of time. With this in mind, any dolphin with less than 240 minutes of time visible during both five-week data collection periods of the study were excluded from analysis. Any individual with more than 240 minutes of time visible in both five-week periods had the second five-week period dropped from analysis. Dolphins with only one five-week period with more than 240 minutes of time visible had that round retained for analysis. The reason the second five-week data were dropped even when meeting criteria was due to rows being excluded during analysis for any missing data. Dolphins without matching data would have been excluded during analysis decreasing the sample size further. These decisions were made to have the largest possible sample size, greatest number of facilities, and to ensure the validity of the results. The thresholds selected would have ensured all dolphins in the final sample would have had at least 10 observations and a majority of the total time filmed. An independent t-test was used to ensure the resulting sample size was not significantly different from the original sample based on age. A chi-square test of significance was used to ensure resulting samples size was not significantly different from the original sample based on sex or habitat type.

### Independent variables

Independent variables were selected to examine a variety of exhibit characteristics and management factors that could impact animal welfare. These variables were created from a survey filled out by animal care staff at participating facilities to provide details on habitat characteristics, environmental enrichment program, and animal training program for the dolphins in this study. All independent variables and definitions are listed in Table 3. Any calculations for independent variables that were necessary and not taken directly from management or trainer surveys are presented by Lauderdale *et al*. [46].

**Table 3:** Independent variables included in the analysis.

Variable	Definition	Values	Type of Variable
***Demographic***			
Sex	Sex of the dolphin	Male/Female	Factor
Age	Age of the dolphin	Years	Covariate
***Environmental Enrichment***			
Enrichment Diversity Index	Enrichment diversity index was created using the Shannon diversity index on the average number of days each enrichment is provided at the facility	Index	Covariate
Enrichment Program Index	Enrichment program index is a standardized factor score created with scores on frequency of enrichment program components used at the facility using a polychoric PCA	Index	Covariate
Night Time Enrichment	Mean number of nights in a week that enrichment was provided to the dolphins at the facility	Number of Nights	Covariate
Enrichment Schedule	Categorical value indicating how enrichment was scheduled at the facility	Predictable, Semi-Random, Random	Factor
Frequency of New Enrichment	Categorical frequency that a facility provided the dolphins with new types/forms of enrichment	Weekly/Monthly, Twice a Year, Yearly/Year+	Factor
***Training***			
Dolphin Presentations	Mean number of dolphin presentations an individual dolphin participated in each week	Mean Number of Presentations	Covariate
Interaction Programs	Mean number of dolphin interaction programs an individual dolphin participated in each week	Mean Number of Interactions	Covariate
Training Duration	Mean amount of time each dolphin interacted with an animal care professional for presentations, interaction programs, training sessions, research, or other training activities each week	Hours	Covariate
Maximum Number of Interaction Guests	Maximum number of participants allowed for an interaction program for that facility	Number of Participants	Covariate
Training Schedule	Categorical variable indicating if the training schedule for the dolphins at that facility was predictable or semi-predictable	Predictable, Semi-Predictable	Factor
***Habitat Characteristics***			
Day Time Spatial Experience	Proportionate volume of water the dolphin had access to based on the percentage of daytime hours spent in different habitats in each five-week data collection period	Megaliter	Covariate
Night Time Spatial Experience	Proportionate volume of water the dolphin had access to based on the percentage of night time hours spent in different habitats in each five-week data collection period	Megaliter	Covariate
24 Hour Spatial Experience	Proportionate volume of water the dolphin had access to based on the percentage of hours throughout the entire day spent in different habitats in each five-week data collection period	Megaliter	Covariate
Length	The maximum straight length in any direction across any habitat the dolphin had access to in each five-week data collection period	M	Covariate
Depth	The maximum depth for any habitat the dolphin had access to in each five-week data collection period	M	Covariate
Habitat Type	Categorical variable indicating the dolphin was in a professionally managed zoo/aquarium habitat or professionally managed ocean habitat	Zoo/Aquarium, Ocean	Factor
Number of Habitats	Maximum number of habitats (different enclosures) dolphin had access to in daytime hours during each five-week data collection period	Number of Habitats	Covariate
Social Management	Categorical variable indicating the type of social management practice for a dolphin during each five-week data collection period	Same Group, Split/Reunited, Rotated Subgroups	Factor
Neighboring Conspecifics	Categorical variable indicating if the dolphin had visual and auditory access to other dolphins without possibility of physical contact during each five-week data collection period	No, Yes	Factor

### Statistical analysis

Descriptive statistics for all independent variables are provided in S1 Data. Given the non-normal distribution of the data, all statistical models were examined using generalized estimating equations (GEE) in SPSS Version 27. GEE can be used when data are not normally distributed and don’t require transformations which preserves the interpretability of the results [47, 48]. For all models, the individual dolphin was used as the unit of analysis while controlling for individual facilities and any significant demographic variables (age and sex). Initial models were examined using only one independent variable at a time, and then subsequent models were built based on those results. Only univariate models where *p* < 0.15 were used to develop and examine the multivariate models [49, 50]. Final models were selected based on significant independent variables and the best quasi-likelihood under the independence model criterion (QIC) [49, 51]. The final models that were considered with significant independent variables and the lowest QIC values are in S2 Data.

## Results

The resulting sample size included 47 dolphins from 25 different facilities that met the minimum criteria for inclusion based on minutes visible. Specifically, there were 19 females and 28 males ranging from 4 to 47 years of age at the start of the study (average 19.68 ± 12.39 SD). There were no significant differences between original and resulting samples based on sex (χ^2^(1, N = 133) = 0.01, *p* > 0.05), age (t (131) = -1.105, *p* > 0.05), or habitat type (χ^2^(1, N = 133) = 3.016, *p* > 0.05). The dolphins include 43 common bottlenose dolphins (91.3%) and 4 Indo-Pacific bottlenose dolphins (8.7%). Behavioral diversity for the sample of dolphins ranged between 0.11 and 1.45 while route tracing behavior ranged between a rate of 0.00 and 0.03 per minute visible. While a variety of independent variables were examined in regard to their univariate relationship with both dependent variables (Tables 4 and 5), eight variables were considered for the multivariate model for behavioral diversity and five variables were examined for route tracing (Tables 6 and 7).

**Table 4:** Summary of univariate analysis for relationships between independent variables and behavioral diversity.

Variables	Reference	N	Beta	*p*-value
***Demographic***				
Sex	Ref = Male	28	0.000	
	Female	19	0.067	0.424
Age		47	-0.008	0.030*
***Environmental Enrichment***				
Enrichment Diversity Index		47	0.098	0.074^
Enrichment Program Index		47	0.029	0.434
Night Time Enrichment		47	-0.004	0.789
Enrichment Schedule	Ref = Predictable	14	0.000	
	Semi-Random	28	0.057	0.561
	Random	5	0.062	0.654
Frequency New Enrichment	Ref = Year+ / Yearly	7	0.000	
	Twice a Year	14	0.415	0.000*
	Monthly /Weekly	28	0.209	0.026*
***Training***				
Dolphin Presentations		47	-0.003	0.488
Interaction Programs		47	-0.001	0.838
Training Duration		47	-0.010	0.265
Maximum Number Interaction Guests		47	0.004	0.450
Training Schedule	Ref = Predictable	16	0.000	
	Semi-Predictable	31	-0.149	0.076^
***Habitat Characteristics***				
Day Time Spatial Experience		47	-0.029	0.700
Night Time Spatial Experience		47	-0.003	0.944
24 Hour Spatial Experience		47	-0.029	0.626
Length		47	-0.003	0.319
Depth		47	0.014	0.120^
Habitat Type	Ref = Zoo/Aquarium Habitat	36	0.000	
	Ocean Habitat	11	-0.109	0.186
Number of Habitats		47	0.081	0.000*
Social Management	Ref = Same Group	17	0.000	
	Split / Reunited at Night	21	0.232	0.004*
	Rotated Subgroups	9	0.322	0.010*
Neighboring Conspecifics	Ref = No Visual Access	31	0.000	
	Visual / Auditory Access	16	0.169	0.062^

^*p* value < 0.15 utilized as significance level for variable selection

*p value < 0.05

**Table 5:** Summary of univariate analysis for relationships between independent variables and route tracing.

Variables	Reference	N	Beta	*p*-value
***Demographic***				
Sex	Ref = Male	28	0.000	
	Female	19	-0.002	0.190
Age		47	0.000	0.418
***Environmental Enrichment***				
Enrichment Diversity Index		47	0.002	0.074^
Enrichment Program Index		47	0.000	0.644
Night Time Enrichment		47	0.000	0.382
Enrichment Schedule	Ref = Predictable	14	0.000	
	Semi-Random	28	0.002	0.181
	Random	5	0.002	0.345
Frequency New Enrichment	Ref = Year+ / Yearly	7	0.000	
	Twice a Year	14	0.002	0.060^
	Monthly / Weekly	28	0.003	0.019*
***Training***				
Dolphin Presentations		47	0.000	0.801
Interaction Programs		47	0.000	0.401
Training Duration		47	0.000	0.945
Maximum Number Interaction Guests		47	0.000	0.358
Training Schedule	Ref = Predictable	16	0.000	
	Semi-Predictable	31	0.000	0.909
***Habitat Characteristics***				
Day Time Spatial Experience		47	0.002	0.117^
Night Time Spatial Experience		47	0.002	0.017*
24 Hour Spatial Experience		47	0.002	0.051^
Length		47	0.000	0.646
Depth		47	0.000	0.824
Habitat Type	Ref = Zoo/Aquarium Habitat	36	0.000	
	Ocean Habitat	11	-0.001	0.658
Number of Habitats		47	0.000	0.561
Social Management	Ref = Same Group	17	0.000	
	Split / Reunited at Night	21	0.001	0.443
	Rotated Subgroups	9	0.004	0.206
Neighboring Conspecifics	Ref = No Visual Access	31	0.000	
	Visual / Auditory Access	16	0.002	0.288

^*p* value < 0.15 utilized as significance level for variable selection

*p value < 0.05

**Table 6:** Descriptive statistics for independent variables considered for multi-variate model of behavioral diversity.

Independent Variables	Reference	n	Mean	SD	Min	Max	Median
Age	-	47	19.68	12.45	4.00	47.00	15.00
Enrichment Diversity Index	-	47	1.74	0.65	0.00	2.59	1.89
Frequency New Enrichment	Ref = Year+ / Yearly	7	-	-	-	-	-
	Twice a Year	14	-	-	-	-	-
	Monthly / Weekly	28	-	-	-	-	-
Training Schedule	Ref = Predictable	16	-	-	-	-	-
	Semi-Predictable	31	-	-	-	-	-
Depth (m)	-	47	9.04	4.97	3.43	19.20	7.90
Number of Habitats	-	47	3.66	1.82	1.00	9.00	4.00
Social Management	Ref = Same Group	17	-	-	-	-	-
	Split/Reunited	21	-	-	-	-	-
	Rotated Subgroups	9	-	-	-	-	-
Neighboring Conspecifics	Ref = No Visual Access	31	-	-	-	-	-
	Visual / Auditory Access	16	-	-	-	-	-

**Table 7:** Descriptive statistics for independent variables considered for multi-variate model of route tracing.

Independent Variables	Reference	n	Mean	SD	Min	Max	Median
Enrichment Diversity Index	-	47	1.74	0.65	0.00	2.59	1.89
Frequency New Enrichment	Ref = Year+ / Yearly	7	-	-	-	-	-
	Twice a Year	14	-	-	-	-	-
	Monthly / Weekly	28	-	-	-	-	-
Day Time Spatial Experience (ML)		47	0.97	0.62	0.12	2.39	0.91
Night Time Spatial Experience (ML)		47	1.34	0.87	0.38	3.92	1.07
24 Hour Spatial Experience (ML)		47	1.21	0.65	0.38	2.61	0.93

Independent variables considered for the multivariate model examining behavioral diversity included age (demographic), enrichment diversity index and frequency of new enrichment (environmental enrichment), training schedule (animal training), depth, number of habitats, social management, and neighboring conspecifics (habitat characteristics). Independent variables considered for the multivariate model examining route tracing included enrichment diversity index and frequency of new enrichment (environmental enrichment), and day time spatial experience, night time spatial experience, and 24-hour spatial experience (habitat characteristics). The final multivariate model for behavioral diversity included enrichment diversity, training schedule, habitat depth, number of habitats, and social management (Table 8). There was a significant inverse relationship between behavioral diversity and enrichment diversity (β = -0.186, *p* = 0.001). Dolphins that were trained on a semi-predictable schedule had lower behavioral diversity when compared to dolphins trained on a predictable schedule (β = -0.364, *p* < 0.001). There was a significant inverse relationship between behavioral diversity and depth (β = -0.021, *p* = 0.013), and dolphins had higher behavioral diversity if managed as split and united groups (β = 0.164, *p* = 0.010) or rotated subgroups (β = 0.361, *p* < 0.001) when compared to animals managed in the same group throughout the day. The final multivariate model for route tracing included enrichment diversity and night time spatial experience (Table 9). There was a positive significant correlation between route tracing and enrichment diversity (β = 0.002, *p* = 0.036), and route tracing and night time spatial experience (β = 0.002, *p* = 0.010).

**Table 8:** Results from multi-variate model examining behavioral diversity (*p < 0.05).

Variable	Beta	95% Confidence Interval	*p*-value
Intercept	0.797	0.551–1.044	<0.001*
Enrichment Diversity	-0.186	-0.299 - -0.073	0.001*
Training Schedule: Predictable	-		
Training Schedule: Semi-Predictable	-0.364	-0.501 - -0.226	<0.001*
Depth	-0.021	-0.038 - -0.004	0.013*
Number of Habitats	0.142	0.101–0.182	<0.001*
Social Management: Same Group			
Social Management: Split/United	0.164	0.039–0.288	0.010
Social Management: Rotated Subgroups	0.361	0.166–0.557	<0.001*

**Table 9:** Results from multi-variate model examining route tracing (*p < 0.05).

Variable	Beta	95% Confidence Interval	p-value
Intercept	-0.003	-0.007–0.000	0.084
Enrichment Diversity	0.002	0.000–0.004	0.036*
Night Time Spatial Experience	0.002	0.000–0.003	0.010*

## Discussion

Results from the current study suggest there are multiple ways to potentially increase behavioral diversity across accredited facilities. Similar to previous studies with elephants [2], it appears that management practices (e.g., social management and training programs) may have a greater influence on dolphin welfare than the size of the habitats based on the significant variables in the final models. Significant relationships for behavioral diversity included enrichment diversity, training schedules, depth, number of habitats, and social management. Alternatively, route tracing, a form of stereotypic behavior, had fewer significant predictors with only enrichment diversity and night time spatial experience related to this behavior.

While behavioral diversity has not been validated as a positive indicator of welfare, there is substantial evidence suggesting it may be a good indicator of positive welfare for many species including dolphins [13, 31]. Previous research has demonstrated that behavioral diversity tends to be higher following management changes thought to enhance welfare [34, 52]. However, in the current study, the opposite was observed where higher enrichment diversity was related to lower behavioral diversity. Similarly, there was also a positive relationship between enrichment diversity and route tracing. Neither of these findings would be expected based on previous research on environmental enrichment [31, 53]. While a significant positive relationship was observed between route tracing and enrichment diversity, this could possibly be explained by animal care staff providing more diverse enrichment in an attempt to decrease the behavior as opposed to enrichment eliciting the behavior. Previous research has demonstrated that providing environmental enrichment can be successful in reducing stereotypic behavior [53] so it is encouraging to see facilities providing a diversity of enrichment likely trying to decrease route tracing. Given the low rates of stereotypic behavior observed, enrichment diversity likely was not causing route tracing as higher levels would have been expected in the current study. In addition, these findings might be attributed to the inverse relationship that is observed between route tracing and behavioral diversity in bottlenose dolphins [13]. However, it is important to note that route tracing in the current study did not include swimming in a circular pattern due to the shape of a habitat, as that would not be considered a stereotypic or abnormal repetitive behavior. Specifically defining a behavior such as route tracing for scientific examination is critical given appropriate forms of pattern swimming with conspecifics observed in bottlenose dolphins under professional care.

The only animal training variable remaining in the final model was dolphins displaying higher behavioral diversity when trained on a predictable schedule when compared to a semi-predictable schedule. In other words, dolphins trained at identical times each day had higher behavioral diversity than animals trained on a more variable time schedule. A review conducted on the predictability of favorable events for animals under professional care suggested that an unpredictable temporal schedule is favorable as long as cues were predictable [54]. However, the authors also noted that the literature only covered a limited number of species and taxonomic differences could exist. Another study from the Cetacean Welfare Study found bottlenose dolphins had higher energy expenditure when trained on a predictable schedule when compared to a semi-predictable schedule [55]. It is possible, more predictable temporal schedules of training sessions allow animals to be more aware of when sessions occur and can spend more time outside of sessions engaged in a more diverse array of behavior and spending more energy in normal physical activity. Previous research has demonstrated the importance of these types of sessions for dolphins [56] and that daily schedules can influence behavior of dolphins when outside of training sessions [57]. Results from the current study suggest that animal care staff should continue to observe the dolphins outside of formal training sessions on a regular basis to determine what is best for individual animals in relation to the predictability of positive events such as training sessions.

As previously noted, management factors may be more important to the welfare of dolphins than characteristics of the habitats. The only variables related to the size of the habitat were depth and night time spatial experience. Depth had a significant inverse relationship with behavioral diversity and night time spatial experience had a significant positive relationship with route tracing. While it would not be recommended to decrease the size of current habitats, smaller habitats in the current study do not seem to relate to decreased behavioral diversity or increased stereotypic behavior. Previous research has suggested that bottlenose dolphins, even when provided with deeper locations within a habitat, choose to spend time in moderate depths of water [27]. While we do not think this is likely due to the stringent criteria used for selecting the final sample of dolphins due to visibility, the significant relationship observed between behavioral diversity and depth could be an artifact of coding the videos. It is possible that the behavior of the dolphins in deeper habitats was more difficult to observe and score.

The total number of habitats was positively related to behavioral diversity. Research with other species has shown that having the ability to distance self both physically and visually from other animals, can lead to positive welfare. For example, increasing the number of climbing structures and hiding places in clouded leopard (*Neofelis nebulosi*) habitats led to a decrease in fecal cortisol metabolite levels [58]. Similarly, leopard cats (*Prionailurus bengalensis*) with additional hiding places were exhibited less pacing behavior and had lower urinary glucocorticoid metabolite concentrations [59]. Perhaps for bottlenose dolphins, a species with a recognized dominance hierarchy [60], having the ability to distance themselves physically from other group members may be important.

A final significant relationship observed was dolphins that were managed in groups that were split and reunited or rotated through subgroups had higher behavioral diversity when compared to dolphins managed in the same group. As bottlenose dolphins are a complex social species that live in a fission-fusion society, the split and reunited and rotated subgroup methods of social management may more closely mimic their natural history [61]. However, with their dominance hierarchy [60], it would be important to make sure that animals that are rotated are socially compatible so as not to increase rates of aggression that have been previously observed to be quite low for bottlenose dolphins under professional care [62].

The limitation of the current work is unfortunately the lowered sample size due to turbidity in open ocean pens and glare reducing visibility of the animals. Even with the limitation, this is the largest multi-institutional study examining the welfare of cetaceans. Additionally, we were unable to control for subspecies due to the low number of Indo-Pacific bottlenose dolphins but investigating subspecies differences would be an interesting topic of future research. The combination of both significant and non-significant relationships observed with independent variables start to provide some additional knowledge on factors that influence bottlenose dolphin welfare. While stereotypic behavior does not always equate to compromised welfare, it should always be examined to try and determine the cause. In the current study, the increased enrichment diversity provided by animal care professionals may be a reaction to the stereotypic or repetitive behavior as opposed to the cause. While route tracing occurred at very low levels in the current study as well as in previous research [10], future efforts should aim to better understand this behavior. There were variables found to be significantly related to behavioral diversity providing a roadmap of potential management changes that could be made to help ensure the behavioral needs of dolphins under professional care are being met. As the current research is correlational in nature, future research using an experimental design could help confirm the impact of making animal management changes. Focusing on management characteristics could help ensure each individual dolphin has the opportunity to thrive.

## Supporting information

S1 Data Descriptive statistics. (XLSX)

S2 Data Model selection. (XLSX)

S1 Appendix Miller et al BehavioralIndicators. (XLSX)
